# Predictors for Extending Hospitalization Stay in Electroconvulsive Therapy Recipients With Bipolar Disorder, Manic Episodes

**DOI:** 10.7759/cureus.8832

**Published:** 2020-06-25

**Authors:** Krishna Priya Bodicherla, Keerthika Mathialagan, Emmanuelle J Caraballo-Rivera, Rikinkumar S Patel

**Affiliations:** 1 Psychiatry, Sri Devaraj Urs Medical College, Kolar, IND; 2 Psychiatry, Sree Balaji Medical College and Hospital, Chennai, IND; 3 Medicine, Ross University School of Medicine, Bridgetown, BRB; 4 Psychiatry, Griffin Memorial Hospital, Norman, USA

**Keywords:** ect, electro-convulsive therapy, bipolar disorders, manic episodes, length of stay, inpatient care

## Abstract

Objectives

We aim to discern the demographic predictors that may extend the hospitalization length of stay (LOS) for patients with bipolar disorder (BD), manic episodes managed with electroconvulsive therapy (ECT), and to study the impact of insurance and hospital characteristics on LOS.

Methods

We used the Nationwide Inpatient Sample (NIS, 2012-2014) from the United States hospitals and included 2,785 adult inpatients (mean age 51.3 ± 16.2 years) with a primary diagnosis of BD, manic episode, and managed with ECT. The median LOS of the sample population is 16 days, and the study inpatients were divided into subgroups: ≤16 days versus >16 days. The logistic regression model was used to find the odds ratio (OR) for the associations of demographic and hospital variables with inpatient stay >16 days versus ≤16 days.

Results

BD inpatients managed with ECT during their hospitalization had a mean LOS of 21.6 ± 22.1 days. About 48.65% (N = 1355) had LOS >16 days. Older adults (age >50 years) have 2.4 times higher odds (95% CI 2.06-2.87) for hospital LOS >16 days compared to younger adults. Although a higher proportion of females received ECT (71.8%), males had two times higher odds (95% CI 1.59-2.27) for hospital LOS >16 days. BD inpatients covered by private insurance/self-pay were at 1.5 times higher odds (95% CI 1.27-1.77) for hospital LOS >16 days. In terms of hospital setting, ownership type and teaching status are significant predictors with inpatients managed in public and teaching hospitals at higher odds for LOS >16 days.

Conclusions

Older men and inpatients covered by private insurance/self-pay have a higher likelihood of extended hospitalization stay during ECT management of BD, manic episodes. The LOS is also influenced by hospital setting with patients managed in public teaching hospitals at higher odds of longer LOS compared to their counterparts.

## Introduction

Bipolar disorder (BD) is a disabling mental illness with episodes of severe mood disturbance associated with significant morbidity and mortality. The World Health Mental Survey Initiative reported a total lifetime BD prevalence of 2.4% worldwide, but the prevalence of BD in the United States (US) was 1% higher than the rest of the world [1,2]. The first episode of bipolar mania has an annual incidence of five per 100,000 of the population [2]. About 53%-55% of BD patients are young adults (age 18-35 years) and females, and two-thirds are whites [3]. There exists a higher burden of both medical and psychiatric comorbidities in the BD population with women at a higher risk of migraine, hypothyroidism, and inflammatory conditions, such as asthma, Crohn's disease, and multiple sclerosis [3,4].

According to the Canadian Network for Mood and Anxiety Treatments (CANMAT) and the International Society for Bipolar Disorders (ISBD) 2018 guidelines, first-line treatment for BD, manic episodes includes combination management with atypical antipsychotics (quetiapine, aripiprazole, risperidone, or asenapine) and lithium or divalproex [5]. Electroconvulsive therapy (ECT) is efficacious in acute mania, and in treatment-resistant patients, with response rate ranging from 80% to 90% [6]. However, despite the high response rate, ECT is considered a second-line treatment for BD, manic episodes as it is considered as a "last resort" approach [5].

ECT is one of the oldest and most effective treatment modalities available for psychiatric illnesses. Recently, the US Food and Drug Administration (FDA) reclassified ECT from class III to lower risk category II; yet ECT is "last resort treatment" when conservative methods have failed for adults with severe mood disorders [5,7]. Indications for ECT depend on the diagnosis (severe, major depression/mania with or without psychotic features), severity of symptoms (life-threatening symptoms such as refusal to eat or drink, severe suicidality, uncontrollable mania, and florid psychosis), and lack of treatment response (failure to respond to at least two adequate trials of psychotropic medications) [8]. Lower utilization rate of ECT in BD patients may be attributed to higher prevalence and risk of delirium and cognitive side effects when ECT is combined with mood stabilizers like lithium [9].

We aim to utilize the Nationwide Inpatient Sample (NIS) to discern the demographic predictors that may extend the hospitalization length of stay (LOS) for patients with BD, manic episodes managed with ECT. Next is to study the impact of insurance and hospital characteristics (including ownership, teaching status, bed size, and location) on LOS.

## Materials and methods

We used the NIS from 2012 to 2014 which is a part of the Healthcare Cost and Utilization Project (HCUP). The NIS is the largest publicly available de-identified database and does not require institutional review board approval. It contains inpatient data from a sample of 4,411 hospitals across 44 states in the US [10].

Demographic variables included were age (18-50 and >50 years), sex (male and female), race (white and non-white), and primary payer (private/self-pay and public). Hospital variables drawn from the American Hospital Association (AHA) Annual Survey of Hospitals included ownership (public and private), bed size (small/medium and large), and location (urban and rural). A hospital was also grouped based on teaching status if it has one or more Accreditation Council for Graduate Medical Education (ACGME) approved residency program, or is a member of the council of teaching hospitals. We evaluated the LOS which was calculated as the number of nights the patient was inpatient for the treatment of BD, manic episode [11].

In our study, we included 2,785 adult inpatients (age ≥18 years) with a primary discharge diagnosis of BD, manic episode using the International Classification of Diseases, Ninth Revision (ICD-9) diagnosis codes: 296.00-296.06, 296.10-296.16, 296.40-296.46, 296.60-296.66, or 296.81, and managed with ECT (ICD-9 procedure code, 94.27) [12]. We found the median LOS of the sample population, i.e. 16 days (mean 21.6 days), and divided into subgroups: inpatient ≤16 days versus >16 days.

We used descriptive statistics to determine differences in subgroups by patients’ demographic and hospitals’ characteristics. Pearson’s chi-square test and independent-sample t-test were used for categorical and continuous data respectively. The logistic regression model was used to find the odds ratio (OR) for the association of demographic and hospital variables with inpatient stay >16 days versus ≤16 days. We used the statistical package for the social sciences (SPSS) version 26 (IBM Corporation, Armonk, NY) to conduct all data analyses, and the statistical significance was set at ≤0.05.

## Results

Our adult inpatients (mean age 51.3 ± 16.2 years) managed primarily for BD, manic episode with ECT during their hospitalization with a mean LOS of 21.6 ± 22.1 days. Major proportion of study inpatients were females (71.8%) and whites (78.5%).

Out of 2,785 total inpatients, 48.65% (N = 1355) had LOS >16 days. These inpatients were mainly older adults (age >50 years) (62.4%), females (66.8%), and whites (70.5%). A higher proportion of inpatients >16 days were covered by public insurances, including Medicare and Medicaid (63.9%) managed in private (89.7%), large bed size (82.4%), urban (98.5%), and teaching (82.7%) hospitals (Table 1).

**Table 1:** Demographic and hospital-wise distribution of bipolar disorder, manic episode inpatients LOS: length of stay

Variable	LOS ≤16	LOS >16	Total	P-value
Total N	1,430	1,355	2,785	-
Age at admission, %				
18–50 years	56.3	37.6	47.2	<0.001
+50 years	43.7	62.4	52.8	
Sex, %				
Male	23.4	33.2	28.2	<0.001
Female	76.6	66.8	71.8	
Race				
White	86.4	70.1	78.5	<0.001
Non-white	13.6	29.9	21.5	
Insurance				
Private/self-pay	43.0	28.8	36.1	<0.001
Public	57.0	71.2	63.9	
Hospital ownership type				
Public	4.9	10.3	7.5	<0.001
Private	95.1	89.7	92.5	
Hospital bed size				
Small/medium	18.2	17.0	17.6	0.403
Large	81.8	83.0	82.4	
Hospital location				
Rural	1.0	1.5	1.3	0.311
Urban	99.0	98.5	98.7	
Hospital teaching status				
Non-teaching	23.1	17.3	20.3	<0.001
Teaching	76.9	82.7	79.7	

Older adults (age >50 years) have 2.4 times higher odds (95% CI 2.06-2.87) for hospital LOS >16 days compared to younger adults (age <50 years). Although a higher proportion of females received ECT management for BD, manic episodes, in the regression model we found that males had two times higher odds (95% CI 1.59-2.27) for hospital LOS >16 days. On the contrary, ECT was utilized mainly in whites and they were at lowers odds (OR 0.35, 95% CI 0.28-0.42) for extended hospitalization of LOS >16 days. Inpatients covered by private insurance or self-payment were at 1.5 times higher odds (95% CI 1.27-1.77) for hospital LOS >16 days. In terms of hospital characteristics, ownership type and teaching status are significant predictors with inpatients managed in public hospitals at two times higher odds (95% CI 1.39-2.64) and teaching hospitals at 1.4 times higher odds (95% CI 1.12-1.68) for LOS >16 days compared to private hospitals (Table 2).

**Table 2:** Predictors of length of stay above 16 days in bipolar disorder, manic episode inpatients

Variable	Odds ratio	95% confidence interval		P-value
		Lower	Upper	
Age at admission				
18–50 years	Reference			
+50 years	2.43	2.06	2.87	<0.001
Sex, %				
Male	1.89	1.59	2.27	<0.001
Female	Reference			
Race				
White	0.35	0.28	0.42	<0.001
Non-white	Reference			
Insurance				
Private/self-pay	1.49	1.27	1.77	<0.001
Public	Reference			
Hospital ownership type				
Public	1.92	1.39	2.64	<0.001
Private	Reference			
Hospital bed size				
Small/medium	Reference			
Large	1.22	0.99	1.49	0.069
Hospital location				
Rural	1.59	0.75	3.39	0.222
Urban	Reference			
Hospital teaching status				
Non-teaching	Reference			
Teaching	1.37	1.12	1.68	0.003

## Discussion

ECT was more popularly used in older adults (age >50 years), females, and whites with BD, manic episodes. About three-fourths of our study inpatients were covered by public insurances. Our results are supported by past studies that found ECT was used for mood disorders predominantly in females (65.2%) than in males, and one-third of ECT recipients were aged 65 years and older and more than half of the population is covered by Medicare [12-15]. Several factors may be relevant to higher utilization of ECT in the geriatric population, like patients have a lower tolerance to medication owing to age-associated pharmacokinetic changes and increased sensitivity to psychotropic medications [13]. A national register-based observational study from Sweden found that ECT for BD was associated with high response rates compared to those treated by psychotropic medications and the strongest prognostic factor being “high age” and absence of psychiatric comorbidities [16]. This could be one of the possible reasons for older adults >50 years in our study had 2.4 times higher odds for longer hospitalization stay. Although a higher proportion of females received ECT, which was also seen in past studies, we found that males with BD, manic episodes and managed with ECT have higher odds for longer hospitalization stay [12,15]. About 86% of psychiatric inpatients managed with ECT in the US are whites [15]. We found that race is not a significant predictor for ECT utilization in BD, manic episode patients, and was also supported by another inpatient study [12].

As per a recent national study on utilization of ECT in psychiatric inpatients, ECT was the preferred treatment modality in the majority of private (91.6%), larger (62.7%), and teaching (77.2%) hospitals, and about half of the Medicare beneficiaries [15]. A higher proportion of patients with BD, manic episodes were managed with ECT in large bed size, and private teaching hospitals in urban areas [12]. Patel et al. found that in all psychiatric inpatients managed with ECT, the mean LOS was 19.7 days, and the mean total cost per inpatient was $64,064, and when compared region-wise, ECT inpatients in the northeast and west regions of the US had comparatively longer LOS and total cost [15]. The use of ECT during inpatient management of BD was associated with longer LOS which was mostly due delays in starting ECT rather than the duration of treatment as shown in a retrospective study in an inpatient psychiatric facility [17]. Majority of psychiatric inpatients receive ECT as a last resort, but if ECT is started within the first seven hospital days in severely ill and treatment-resistant patients, it can be a cost-effective model with 14.7-day reduction in LOS and a reduction of $41,976 in total cost during inpatient management [12]. We found some of the hospital characteristics that were significantly associated with increasing the hospital LOS above the median stay, including private insurance/self-pay beneficiaries (by 1.5 times), public hospitals (by two times), and teaching hospitals with residency programs (by 1.4 times). Hospital bed size and location did not have a significant impact on ECT inpatients and hospitalization LOS.

This study has few limitations due to the administrative nature of the NIS. Beyond cross-sectional hospitalization-related data, information was not available about the quality of ECT, including electrical stimulus intensity, electrode placement, and number of ECTs required during the hospitalization for BD, manic episodes. Also, the NIS data lack information about the patient outcomes during the post-ECT period. Besides, the data were limited to non-federal community-based hospitals and excluded ECT inpatients at state and county psychiatric hospitals.

## Conclusions

Older men and inpatients covered by private insurance/self-pay have a higher likelihood of extended hospitalization stay during ECT management of BD, manic episode. The LOS is also influenced by hospital characteristics with patients managed in public teaching hospitals at higher odds of longer LOS compared to their counterparts. Early utilization of ECT in non-teaching and private hospitals, and patients covered by public insurance should be considered to increase the utilization of ECT in BD, manic episodes and reduce overall inpatient LOS.
